# First microsatellite markers for the European Robin (*Erithacus rubecula*) and their application in analysis of parentage and genetic diversity

**DOI:** 10.1038/s41598-021-98364-3

**Published:** 2021-09-23

**Authors:** Aleksandra Gwiazdowska, Oliwia Karpińska, Katarzyna Kamionka-Kanclerska, Patryk Rowiński, Hanna Panagiotopoulou, Jan J. Pomorski, Richard K. Broughton, Luis F. P. da Silva, Robert Rutkowski

**Affiliations:** 1 Museum and Institute of Zoology, Polish Academy of Sciences, Wilcza 64, 00-679 Warsaw, Poland; 2 Institute of Forest Sciences, Warsaw University of Life Sciences, Nowoursynowska 159, 02-776 Warsaw, Poland; 3 UK Centre for Ecology and Hydrology, Maclean Building, Benson Lane, Crowmarsh Gifford, Wallingford, OX10 8BB UK; 4 CBIO-InBIO Campus Agrário de Vairão Rua Padre Armando Quintas, nº7, 4485-661 Vila do Conde, Portugal

**Keywords:** Genetics, Genetic markers, Population genetics, Zoology, Animal behaviour, Ecological genetics, Molecular ecology

## Abstract

The European Robin is a small passerine bird associated with woodlands of Eurasia and North Africa. Despite being relatively widespread and common, little is known of the species’ breeding biology and genetic diversity. We used Next Generation Sequencing (NGS) to develop and characterize microsatellite markers for the European Robin, designing three multiplex panels to amplify 14 microsatellite loci. The level of polymorphism and its value for assessing parentage and genetic structure was estimated based on 119 individuals, including seven full families and 69 unrelated individuals form Poland’s Białowieża Primaeval Forest and an additional location in Portugal. All markers appeared to be highly variable. Analysis at the family level confirmed a Mendelian manner of inheritance in the investigated loci. Genetic data also revealed evidence for extra-pair paternity in one family. The set of markers that we developed are proven to be valuable for analysis of the breeding biology and population genetics of the European Robin.

## Introduction

The European Robin *Erithacus rubecula*, a small (c.19 g) passerine bird, is a common and widespread species of wooded habitats in Europe, parts of western Asia and North Africa^1^. Although it is a familiar and moderately well-known species in Western Europe, there is little information about the species’ breeding biology across much of its range, and studies of its population genetics are mostly lacking^2–9^. This absence of genetic studies is probably related to the European Robin’s elusiveness during the breeding season, when nests are notoriously difficult to locate^1^.

Most of the available fragmentary data on the breeding biology of European Robins in Britain comes from highly transformed environments, such as gardens and secondary woodlands^4^. However, these studies may not be representative of the European Robin’s ecology in more natural forests elsewhere in its range. This study bias and difficultly of observing breeding European Robins means that there is no information characterising the species’ typical patterns of behaviour and breeding biology in primaeval forest habitats, to which it was originally adapted^10^.

The European Robin is considered to be socially monogamous and highly territorial in the breeding season, when pairs may have up to three consecutive breeding attempts between spring and early summer. Nests are typically located in recesses or cavities on standing or fallen trees within several meters of the ground, in banks or walls, or under overhanging vegetation on the ground itself; nests generally contain 4–5 eggs that are incubated for around 15 days before hatching altricial nestlings that leave after a further 15 days, becoming independent around 10 days later^1,4,5,11,12^. Despite this basic knowledge there is no information on genetic relatedness or extra-pair paternity within or between broods and their parents.

Molecular methods have made an invaluable contribution to the study of the breeding biology and mating systems of birds^13–17^. These techniques have revealed that extra-partner mating is a much more frequent phenomenon among birds than was previously thought^14,16,18^, and extra-pair nestlings occur frequently even among socially monogamous species^14^. Such studies are important in advancing the understanding of female mate choice and reproductive fitness^17,19,20^. The benefits to females of adopting the extra-pair mating strategy are still debated, and there is no certain evidence that the advantages of such breeding activity outweigh the costs. Conversely, males obviously benefit from this behaviour through the increased reproductive success while engaging in the care of nestlings^17^.

Only three studies have applied molecular techniques to describe population genetics of the European Robin. The genetic structure of a resident population from the Canary Islands, which migrate short distances between breeding and wintering grounds, was characterized using polymorphism of mitochondrial DNA^21^. This study revealed that birds from Gran Canaria island should be considered as a distinct subspecies that is genetically separated from birds on continental Europe and the western Canary Islands. In contrast, analysis of population from the Azores indicated a lack of neutral genetic differences between group of birds from the archipelago and the group from mainland Portugal, suggesting a recent colonization of these islands^22^. Another resident population, from southern Italy, was investigated using cross-amplified microsatellites^23^, which suggested significant genetic differentiation between populations with long- and short-distance migratory behaviour.

The use of cross-amplified microsatellites in genetic studies is known to have several potential disadvantages, including particular markers being less polymorphic in target species than in the species of origin^24–26^. Also, diversification of the species may lead to mutations in sequences of flanking regions of some alleles, preventing the primer annealing during PCR^27,28^. This, in turn, may result in a high frequency of ‘null alleles’. Moreover, the success rate of amplification frequently decreases in proportion with the genetic distance between species^29^.

However, to date, there appears to have been no species-specific microsatellite markers described for the European Robin, including for the Central European population that represents the nominative and highly-migratory subspecies. Furthermore, this knowledge gap compounds the lack of data, supported by genetic information, for the diversity, ecology and biology of the European Robin’s populations in natural forest ecosystems across Europe. Such studies from primary forest conditions are informative and highly valuable as a benchmark for understanding a species’ biology in modified ecosystems and more outlying populations in the species’ range.

In this study we provide the first genetic data for European Robins from the last significant expanse of European temperate primaeval forest, the Białowieża Forest in north-eastern Poland, and from a second novel location on the western margin of the species’ European range, in Portugal. Information from the Białowieża Forest provides a unique opportunity to extend our knowledge of the European Robin’s biology in the natural, unmodified forest that still retains an almost complete suite of flora and fauna^30^. Such data provides an important reference system for further studies of evolutionary and ecological processes in this forest, in addition to those occurring in managed forests under strong anthropogenic pressure.

The main aim of the study is to describe the species-specific microsatellite markers for the European Robin, as a resource for population genetics and breeding biology studies. We then apply these molecular markers to investigate the paternity of multiple broods and individuals of European Robins from the Białowieża Forest, and additional samples from individuals resident approximately 2730 km away in Portugal, to estimate genetic diversity of the species across this geographical span.

## Materials and methods

This study was carried out in compliance with the ARRIVE guidelines. All methods were performed in accordance with the relevant guidelines and regulations. The methods used for animal care and handling were approved by 2nd Ethical Committee for Animal Experimentation at Warsaw University of Life Sciences, Poland (decision number: WAW2/93/2017, dated on 15.12.2017) and Portuguese Institute for Conservation, Nature and Forests (ICFN, decision number: 347/2020/CAPT).

### Sample collection and DNA extraction

**Figure 1:**
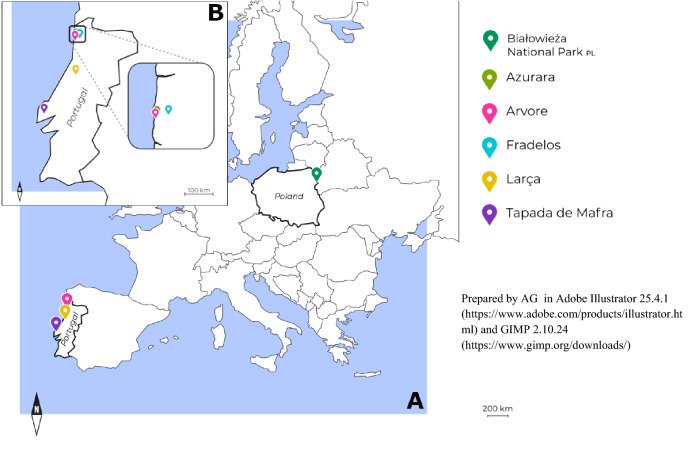
Study area in Poland and Portugal (**A**). Figure (**B**) shows five places where robins were caught to collect samples.

Samples of genetic material from European Robins were collected in two widely separated areas of Europe, in order to isolate microsatellite markers and investigate relatedness. In north-east Poland, birds were sampled between 2018 and 2019 in the Strict Reserve of Białowieża National Park (52° 46′ 0″ N, 23° 52′ 0″ E) (Fig. 1.), which is characterized by features typical of pristine forest, namely a multi-storey profile of tree stands, abundant dead wood with many uprooted trees, tall tree heights and a diverse tree community of deciduous and coniferous species^10^. Genetic material was collect on three sample plots (about 30 ha each) at representative locations in the forest.

European Robins are summer migrants to the Białowieża forest, arriving in early spring in order to breed, after wintering in southern or western Europe. During the breeding season (late March to July), 95 individuals from the Białowieża forest were sampled for genetic analysis. Adult birds (*N* = 19: 11 males, 8 females) were caught using mist-nets (fine sheets of netting suspended between poles) and a recording of songs and calls as a lure. Further samples of a single chick (8–10 days old) from each of 28 nests were also collected. These combined samples enabled genetic analysis for 47 presumably unrelated birds. Additionally, we collected samples from seven ‘full’ families (social father and mother plus all the nestlings) to verify Mendelian inheritance of the isolated microsatellites. These seven ‘full families’ included 14 of the 19 adult birds reported above. The material for genetic analysis was collected during standard capture and marking (with numbered metal leg-rings) procedures for adult birds, and during nest inspections for nestlings, and involved plucking a single tail feather from each individual. Samples were stored in Eppendorf tubes filled with 96% pure ethyl alcohol.In Portugal, where the breeding population of European Robins is resident all year round^31^, 24 samples of adult birds were collected from between April and June 2020 at five locations: (A) Fradelos (41° 21′ 58″ N, 8° 35′ 55.″ W); (B) Azurara (41° 21′ 02″ N, 8° 43′ 50″ W); (C) Arvore (41° 19′ 45″ N, 8° 43′ 59″ W); (D) Larça (40° 18′ 46″ N, 8° 24′ 13″ W); and (E) Tapada de Mafra (38° 57′ 40″ N, 9° 17′ 39″ W) (Fig. 1.).

Fradelos, Azurara and Arvore consist of mixed plantations of *Pinus pinaster* and *Eucalyptus globulus* with low management and an understory composed mostly of small native shrubs. Larça is a secondary native forest (tree height usually lower than 10 m) and a dense and diverse understory. These four locations are integrated in a landscape mainly composed by small forest patches, intertwined with agriculture and urbanized areas. Finally, Tapada de Mafra is a fenced property of more than 800 ha, managed for eco-tourism and big-game hunting with a mosaic of open areas and diverse forests dominated by *Quercus* spp. The birds were captured with mist nests placed along dirt tracks or with spring traps baited with mealworms (*Tenebrio molitor*). A blood sample was collected from each bird by puncturing the brachial vein near the joint between the humerus and ulna with a sterile needle (27G), following standard methods^32^. Approximately 75 µl of blood was collected with a capillary tube and then transferred to a 2 ml tube filled with 98% ethanol, which was stored at 4 °C.

For the feather samples, the main source of DNA is cells attached to the base of the calamus (quill) when feathers are in the growth phase^16,24^, or blood clots within the rachis of fully grown feathers^16,33^. Hence, approximately 0.5 cm of the calamus was extracted and cut into smaller pieces using a sterile scalpel on sterile Petri dish. Cut parts of the calamus were then incubated with a Proteinase K overnight at 56 °C.

For samples from Portugal, blood clots were removed from the alcohol and put into the buffer LT (EURx Polska) and incubated with a Proteinase K overnight at 56 °C. We then used a Tissue DNA Purification Kit (EURx Polska) to extract DNA from the calamus and blood following standard protocols. Each extract was analysed using Nanodrop (Thermo Fisher Scientific) to check the purity and concentration of DNA. The extracted DNA was stored at -20 °C.

### Microsatellites typing with Pacific Biosciences RSII

An amount of 7.4 µg of genomic DNA consisting of four equally (20 µl) mixed DNA samples was used for a 2 kb library construction. DNA was fragmented, using Covaris System according to protocol, into sizes between 1.5 and 3 kb, applying the following modifications: intensity was set to 0.2, and time of sonication to 500 s. Fragmented DNA was purified using AMPure PB Beads applying 0.6 × volume ratio.

Subsequently, 750 ng of shared and concentrated DNA was used for library preparation with SMRTbell Template Prep Kit 1.0, applying the online Pacific Biosciences protocol (PacBio). This procedure consisted of: 1. DNA damage and ends repair, 2. blunt ligation of hairpin adaptors at both ends of the DNA fragments, and 3. removal of failed ligation products with *Exo*III and *Exo*VII enzymes. To separate the enzymatic reactions, DNA was purified with 0.6 × volume ratio of AMPure PB Beads. Size distribution of DNA fragments and its integrity was monitored on 0.5% agarose gels during and after completing the procedure of library preparation. The final library was double purified with 0.6 × volume ratio of AMPure PB Beads. Binding Calculator v.2.3.1.1 (PacBio) was applied for protocol generation in order to prepare the library for sequencing applying the DNA/Polymerase Binding Kit P6 V2 and MagBeads reagents. Sequencing was performed on a PacBio RS II sequencer running three SMRT Cells in the 360-min data collection mode.

Data generated through the PacBio sequencing platform was analysed to obtain Reads of Insert (ROI) according to the RS_ReadsofInsert.1 protocol applying default settings. The ROI reads were further analysed with the msatcommander v.1.08^34^ program in order to identify microsatellite sequences and design primers. A threshold of at least five repetitions for tri- and tetra-nucleotide repeats, excluding mononucleotide repeats, was set in this analysis. Primer design parameters including the msatcommander option “combine loci” were as follows: length of the primers 18–22 bp, annealing temperature (Tm) 58 °C–62 °C, GC content 30%–70%, and amplicon product size 80–400 bp with minimum of 20–30 bp DNA sequence flanking from both sides of the tandem repeat of the microsatellite.

### Microsatellite marker selection and testing

Selection of microsatellite markers was performed in several steps. Firstly, based on information obtained for the set of microsatellite sequences, identified in the genome of the European Robin from the above analyses, loci with the highest number of tandem repeats were selected (total number of 281 sequences). In the second step, loci were filtered based on criteria calculated in silico: (1) maximal PCR efficiency, (2) minimal penalty score, (3) no dimer formation, (4) a maximal difference in melting temperature of 2 °C between a primer pair, and (5) sequence length of the microsatellite ranging from 100 to 350 bp. This gave 40 selected microsatellite loci, which were then divided based on product size into three groups: (1) short < 200 bp, (2) medium 200–300 bp, and (3) long > 300 bp. The reason for this division was optimization of designed multiplex PCR.

Finally, for further testing, we selected 11 short loci, 14 medium loci and 15 long loci. These loci were initially tested on 17 unrelated individuals following the method of Schuelke (2000)^35^ and Austin et al. (2011)^36^. Briefly, the forward primers were tailed by a 5′-CACGACGTTGTAAAACGAC sequence tag, which was identical to four fluorescent labelled dyes: 6-Fam, Hex, Tamra and Rox. The PCR reaction mix contained: 1 µl of DNA, 7.5 µl of 2 × Dream Taq Hot Start Green PCR Master Mix (Thermo Fisher Scientific), 0.02 M tagged forward locus specific primer, 0.2 M locus specific reverse primer and 0.2 M fluorescent labelled universal forward primers. The reaction was performed under the following conditions: initial denaturation for 15 min at 95 °C, 35 cycles of 30 s at 95 °C, 50 s at 57 °C, 45 s at 72 °C and final elongation of 5 min at 72 °C. Microsatellite loci were amplified separately and visualised on 1.5% agarose gel. The genotyping analyses was performed using ABI 3500XL Genetic Analyzer (Applied Biosystems) and the PCR product sizes were read using GENEMAPPER v.4.1 software (Applied Biosystems). Based on the results obtained, 17 microsatellite loci were selected for further analysis and design of multiplex panels. These loci were characterized by a lack of amplification artefacts, a high number of alleles and no more than two peaks per locus on chromatograms.

The multiplex PCR reaction mix contained: 1 µl of DNA extract (containing 47–283 ng of DNA in the case of young birds) or 3 µl of DNA extract (containing 1–78 ng of DNA in the case of adult birds), 7.5 µl of PCR Master Mix (QIAGEN), 0.6 µl of the primer mix (forward and reverse for each locus; concentration: 0.85–1 µM) and 4.65 µl or 2.65 µl water for PCR depending on the amount of DNA extract used. This time forward primers were fluorescently labelled on their 5′ end with one of the following dyes: 6-FAM, TAMRA, HEX and ROX (Sigma-Aldrich). The amplification conditions were as follows: initial denaturation for 15 min at 95 °C, 40 cycles of 30 s at 94 °C, 90 s at 57 °C, 90 s at 72 °C and final elongation for 10 min at 72 °C.

Analysis of the amplification of the microsatellite loci in different combinations of multiplex sets was verified using ABI 3500XL Genetic Analyzer. As a size standard we used GeneScan 600 LIZ (Applied Biosystems). The genotyping was performed using a mixture of formamide with LIZ-600 and PCR product, which was denatured for 5 min. at 95 °C. Genotypes were then identified using GENEMAPPER v.4.1.

### Statistical analysis

All statistical analyses were performed on genotypes of 69 presumably unrelated individuals (45 from Poland and 24 from Portugal). For each locus we assessed the number of alleles (A), unbiased expected heterozygosity (H_E_) and observed heterozygosity (H_O_) using GenAlEx version 6.5^37^ and Hardy–Weinberg equilibrium (HWE), and also Genepop on the Web version 4^38,39^. Additionally, the fixation index (F_IS_), allelic richness and F_ST_ were calculated using FSTAT version 2.9.3^40^.

Parameters of usefulness of the selected microsatellites in paternity verification and population genetics studies were also estimated, using Cervus 3.0^41^. Specifically, we calculated the PIC index (polymorphic information content), F(null) (null allele frequency), NE-1P (average non-exclusion probability for the first parent), NE-2P (average non-exclusion probability for the second parent), NE-PP (average non-exclusion probability for a candidate parent pair), NE-I (average non-exclusion probability for identity of two unrelated individuals) and NE-SI (average non-exclusion probability for identity of two siblings). Probability of identity (PI) and probability of identity for siblings (PI_Sibs_) were estimated using GenAlEx version 6.5^37^.

## Results

RSII sequencing of the SMRT library generated 4,330 Mb of raw data covering 192,298 reads for the three SMRT Cells analysed jointly. The average length of inserts in these runs equalled 1,959, 1,873 and 2,997 bp, respectively (with quality ranging between 0.91 and 0.96). The final output length of reads reached 3,800 Mb. Of these sequence sets, 65,162 reads of insert (ROI) were acquired and accumulated with 87,123,089 bp. The average ROI length equalled 1,337 bp and the average number of passes exceeded 16.

The final sequence list consisted of 62,872 DNA sequences that were subjected to microsatellite loci identification. Altogether 1932 microsatellite loci covering tri- or tetra-nucleotide repeats in at least five repetitions were identified. For the a priori chosen parameters of microsatellite selection for amplification testing, primer pairs for 961 loci were successfully designed. From this set, 18 tri-nucleotide and 22 tetra-nucleotide markers (named ER1-ER40), with the number of repeats ranging between 37 and 10, were further chosen for amplification and polymorphism description (Supplementary Table S1) and then deposited in GenBank under accession numbers SRX10929063-SRX10929102.

Agarose gel electrophoresis indicated that 36 out of 40 tested primer pairs (90%) were successfully amplified. The amplification was evaluated as successful if the length of the band was similar to the size of alleles indicated by mstacommander. Genotyping using ABI 3500XL Genetic Analyzer (Applied Biosystems) showed that 23 primer pairs amplified microsatellite fragments, meaning that the peaks on chromatograms were sharp and the score of the peaks was similar to the expected product size. Finally, 17 primer pairs were selected for designing multiplex PCR. In the case of three primer pairs, amplification failed in multiplex reaction, despite applying several combinations. Hence, we finally designed three multiplex panels (Table 1), consisting of 14 primer pairs, as follows: Mix 1 (ER24, ER40, ER28, ER21, ER38, ER15), Mix 2 (ER32, ER7, ER4, ER5) and Mix 3 (ER39, ER13, ER25, ER31).

**Table 1:** The characterization of three multiplex PCR sets for the amplification of 14 microsatellite loci in 69 unrelated samples of European Robin.

Locus	Primer sequence (5′–3′)	Dye	Size range	A	H_o_	H_E_	F_IS_	HWE	F(null)
ER24	F: TGTTGTCACTTTGCACCTGG	TAM	128–185	16	0.594	0.696	0.154*	0.0104	0.079
	R: CTGCTTCACTCCTTGCACAG								
ER40	F: GATTGCAGGGAACGCTGAC	FAM	190–217	10	0.696	0.799	0.136	0.4742	0.071
	R: CAGGTGCTGCTGGAATGTTC								
ER28	F: TGGTTAATGAAGATGACGGCG	HEX	214–283	14	0.826	0.864	0.052	0.1114	0.021
	R: AAGATCCCAGCCTCATTCCG								
ER21	F: ATGGATGCTGTGGGAGTCTC	ROX	225–273	13	0.913	0.889	− 0.020	0.4540	− 0.013
	R: GTGATCACGACAGTCAGCAC								
ER38	F: CGCCTGTGTAAATCTCGCTG	FAM	283–358	12	0.797	0.799	0.010	0.3520	0.003
	R: TTGTTCCAACCTAGCCTGGG								
ER15	F: TACCTCCTGCTTGCTGTGAG	HEX	299–415	21	0.913	0.908	0.002	0.7325	− 0.002
	R: TCCCAACCAAGTCTCCAAGG								
ER32	F: CTCACCTGTGCTCTTTAAGGC	TAM	106–139	9	0.638	0.730	0.133	0.4697	0.066
	R: TGCAGTTCTTCAGTTTGGTGG								
ER7	F: CAACTGGTCAGCAACACTCC	ROX	262–294	10	0.899	0.851	− 0.048	0.7983	− 0.028
	R: TTGCAAGCTCAGGATCCTTG								
ER4	F: TCAGAGGTGTGGCAGAAAGG	FAM	204–292	17	0.696	0.850	0.188	0.0675	0.102
	R: TTGTTCCTGCTGCTTTGGAC								
ER5	F: ACTCCCTACACTTGCCAAGG	FAM	308–428	14	0.826	0.842	0.026	0.7992	0.009
	R: GTCTAAAGATGCCCAGAACCTG								
ER39	F: CGGTGAACAAGAGCAGAAGG	TAM	144–216	22	0.913	0.923	0.018	0.5100	0.004
	R: CCTTCTTCACTGCAAGCTGG								
ER13	F: AGTCTCCTTGCTGCTCTGTG	ROX	180–256	19	0.913	0.885	− 0.025	0.2387	− 0.017
	R: TGCCCTTTCTCTGACATGGG								
ER25	F: GAAAGTATCGTGGCAATGCAC	HEX	272–332	19	0.896	0.914	0.028	0.3522	0.010
	R: TTCAGGTTCGACAAAGAGGC								
ER31	F: GAAATGGGAACTGTGCTGGC	FAM	298–344	26	0.754	0.935	0.201*	0.0000	0.106
	R: CTGCAGGCTTACTTCAACCAG								

Column headings are as follows: Size range—size of fragments obtained during PCR, including flanking region; A—number of alleles; H_O_—observed heterozygosity; H_E_—expected heterozygosity; F_IS_—fixation index (*—F_IS_ value significant after Bonferroni correction, 200 randomizations, adjusted *P*-value = 0.00357), HWE—*P-*value of Hardy–Weinberg equilibrium exact test, F(null)—null allele frequency. Annealing temperature for all PCRs was 57 °C.

We failed to amplify loci ER25 in three samples, but the remaining 13 loci were amplified in all 119 samples. The resulting characterization of microsatellite polymorphism in 69 unrelated individuals of the European Robin indicated that the number of alleles at a single locus ranged from nine (locus ER32) to 26 (locus ER31).

The highest observed and expected values of heterozygosity were found in loci ER21, ER15, ER39, ER13 (H_O_ = 0.913;) and ER31 (H_E_ = 0.935). The lowest value of heterozygosity was observed in locus ER24 (H_O_ = 0,594; H_E_ = 0.696) (Table 1). Two loci (ER24 and ER31) significantly deviated from HWE (Table 1). Accordingly, in these loci, F_IS_ was significantly greater than zero, suggesting heterozygote deficiency. Overall, the observed heterozygosity was high (H_O_ = 0.805) and significantly lower than the expected heterozygosity (H_E_ = 0.849, *P* < 0.001). The estimated frequency of null alleles [F(null)] was very low, reaching 10% only in two cases (Table 1).

Considering the two separate locations, analysis of the 45 individuals from the population from Poland also indicated significant deviation from Hardy–Weinberg Equilibrium due to heterozygote deficiency (H_O_ = 0.819; H_E_ = 0.846, *P* < 0.002), although F_IS_ was low and non-significant after Bonferroni correction (F_IS_ = 0.04; adjusted *P*-value = 0.004, 280 permutations). Similarly, in the group of samples from Portugal the observed heterozygosity was lower than expected (H_O_ = 0.780; H_E_ = 0.820, *P* = 0.063), with an associated low value of F_IS_ (F_IS_ = 0.07). The mean number of alleles was higher in Poland (mean A = 13.64) than in Portugal (mean A = 10.71), although allelic richness was similar in both regions (Portugal *R* = 10.71; Poland *R* = 11.60).

Genetic differentiation, assessed as F_ST_, was low between both regions (F_ST_ = 0.015) but this was statistically significant (*P* < 0.05).

Parameters interlinked with the usefulness of identified loci in the parentage and relatedness analysis indicated that the described microsatellite panels can be successfully used in such studies. The polymorphic information content was high in all loci (PIC > 0.5; Table 2), and, accordingly, the analysis of 69 unrelated individuals showed a very low non-exclusion probability for the combination of 14 loci. The probability of identity and probability of sibling identity was the lowest in locus ER31 (PI = 0.01; PI_Sibs_ = 0.28) and the highest were in loci ER24 (PI = 0.11; PI_Sibs_ = 0.43) and ER32 (PI = 0.11; PI_Sibs_ = 0.41). The respective values for the combination of 14 loci were also very low (Table 2).

**Table 2:** Informativeness and exclusion probability of analyzed loci, estimated based on genotypes of 69 unrelated individuals of European Robin from Poland and Portugal.

Locus	PIC	PI	PI_Sibs_	NE-1P	NE-2P	NE-PP	NE-I	NE-SI
ER24	0.675	0.11	0.43	0.678	0.489	0.272	0.114	0.430
ER40	0.773	0.066	0.37	0.560	0.383	0.196	0.066	0.367
ER28	0.850	0.032	0.33	0.426	0.269	0.107	0.032	0.326
ER21	0.879	0.022	0.31	0.367	0.223	0.078	0.022	0.311
ER38	0.772	0.068	0.37	0.563	0.387	0.202	0.068	0.367
ER15	0.901	0.016	0.3	0.313	0.185	0.055	0.016	0.300
ER32	0.695	0.11	0.41	0.663	0.482	0.287	0.107	0.412
ER7	0.834	0.040	0.33	0.463	0.299	0.131	0.040	0.334
ER4	0.834	0.039	0.33	0.456	0.294	0.124	0.039	0.335
ER5	0.823	0.045	0.34	0.484	0.316	0.146	0.045	0.340
ER39	0.918	0.011	0.29	0.269	0.156	0.040	0.011	0.291
ER13	0.874	0.023	0.31	0.373	0.229	0.080	0.023	0.314
ER25	0.908	0.014	0.30	0.295	0.173	0.049	0.014	0.296
ER31	0.931	0.0079	0.28	0.231	0.131	0.029	0.008	0.285
Overall	0.8334	1.2E−21	2.1E−07	0.00000486	8.737E−0009	1.513E−0014	1.175E−0021	0.00000021

PIC—polymorphic information content, PI—probability of identity; PI_Sibs_—probability of identity for sibs; paternity non-exclusion probability (first second and pair parent, NE-1P, NE-2P and NE-PP respectively) and the combined non-exclusion probability of identity and sibling identity (NE-I and NE-SI) for each marker; overall—values for combination of 14 loci.

Paternity analysis in the seven families (with known social parents) indicated extra-pair paternity in only one case (Table 3, family WE8). In two of the nestlings, we didn’t find the putative father’s alleles in ten out of 14 investigated loci. Moreover, analysis of full families confirmed that all microsatellite loci had Mendelian inheritance (Table 3).

**Table 3:** Microsatellite genotypes of members of three full families of the European Robin.

MIX	MIX 1												MIX 2								MIX 3								Family
Primer	ER24		ER40		ER28		ER21		ER38		ER15		ER32		ER7		ER4		ER5		ER39		ER13		ER25		ER31		
F	140	155	208	214	220	235	249	265	283	295	311	327	118	127	278	286	224	228	316	332	180	186	192	208	302	311	334	343	**MO6**
M	137	137	205	205	217	229	253	257	295	298	303	331	118	121	270	286	216	228	316	328	183	201	196	224	290	305	316	322	
1	137	140	205	208	220	229	257	265	295	295	311	331	118	121	270	278	216	224	316	328	183	186	208	228	290	311	316	343	
2	137	140	205	208	220	229	249	257	295	298	303	327	121	127	270	286	228	228	316	316	183	186	196	208	302	305	316	334	
3	137	140	205	214	217	220	249	257	295	298	303	311	118	118	270	278	216	224	328	332	180	183	208	224	302	305	322	343	
4	137	155	205	214	217	235	249	257	283	298	303	311	121	127	270	286	228	228	328	332	186	201	208	224	290	311	316	334	
5	137	155	205	208	217	235	249	253	283	295	303	311	118	118	278	286	216	224	316	328	183	186	192	196	305	311	322	334	
6	137	155	205	208	217	220	253	265	295	298	303	311	118	121	270	286	216	228	328	332	183	186	196	208	290	311	316	334	
7	137	155	205	208	217	220	253	265	295	295	327	331	121	127	286	286	216	228	316	316	186	201	192	224	302	305	322	334	
8	137	155	205	208	217	235	249	253	283	295	327	331	118	127	278	286	216	224	328	332	183	186	192	196	290	311	316	334	
F	140	140	199	211	220	220	241	257	292	295	303	339	118	121	270	290	224	240	328	388	168	183	192	196	302	323	316	319	**WA6**
M	140	140	193	205	217	220	261	269	292	292	335	339	112	121	278	282	208	228	328	328	186	210	188	192	308	317	319	328	
1	140	140	193	211	217	220	241	269	292	292	335	339	118	121	282	290	208	240	328	328	183	210	188	196	308	323	319	328	
2	140	140	193	211	220	220	257	269	292	295	303	335	118	121	270	278	228	240	328	388	183	210	188	196	317	323	319	328	
3	140	140	193	211	220	220	241	261	292	295	303	335	121	121	270	278	224	228	328	388	183	210	192	196	302	317	319	319	
4	140	140	193	211	217	220	241	261	292	292	335	339	112	118	278	290	224	228	328	328	183	186	188	196	317	323	319	328	
5	140	140	193	211	217	220	241	261	292	292	339	339	112	121	270	278	228	240	328	328	168	210	188	196	317	323	316	319	
F	128	140	199	205	217	232	253	257	289	295	335	339	118	121	274	286	216	224	328	332	147	171	188	248	299	299	325	325	**WE8**
M	140	143	214	214	217	232	261	269	286	292	335	343	118	118	266	274	216	216	324	332	159	168	200	208	0	0	313	319	
1	128	143	199	199	217	217	257	261	286	289	335	343	118	118	274	286	216	216	324	332	147	168	188	208	299	308	319	325	
2	140	140	**199**	**202**	217	232	**257**	**257**	**295**	**295**	**315**	**335**	118	121	286	**290**	**204**	216	328	332	**147**	**171**	**188**	**188**	299	302	**325**	**331**	
3	140	140	199	199	217	232	257	269	286	295	339	343	118	118	274	274	216	216	324	332	147	168	200	248	299	308	313	325	
4	140	143	199	214	217	217	257	269	289	292	335	339	118	118	274	286	216	224	324	332	159	171	188	208	299	308	313	325	
5	140	140	205	205	217	232	253	261	286	289	335	343	118	118	274	274	216	216	324	332	159	171	188	208	275	299	319	325	
6	128	143	199	199	217	217	257	269	286	295	335	343	118	121	274	274	216	224	324	332	159	171	200	248	299	308	319	325	
7	128	140	**199**	**202**	217	232	**253**	**257**	289	295	**315**	**335**	**118**	**133**	286	**290**	**204**	224	324	332	**147**	**171**	**188**	**252**	299	302	**325**	**331**	

Example of families MO6 and WA6 confirm Mendelian manner of inheritance of investigated loci. Family WE8 shows detection of extra-pair paternity within the brood. Genotypes shown in bold suggest genetic contribution of an ‘extra’ male. F—female, M—social male, 1–8—nestlings.

## Discussion

The PacBio RSII sequencing enabled microsatellite markers for European Robins to be discovered in a sufficient number, and the restricted selection using narrow criteria enhanced the possibility of choosing high quality markers for population analysis. This method is often chosen for de novo microsatellite characterization, and is recognised as being reliable and efficient^42–44^. It is a method of choice in cases of non-model organisms with no available genomic data, as well as allowing the obtaining of long reads (here *ca*. 2 kb) that cover the whole microsatellite sequence together with a large flanking region. This method, coupled with a universal primer labelling system^35^, was proven to be highly cost effective and efficient when compared to the traditional approaches of microsatellite description^42,45^, or testing via individual fluorescent labelling of all of the tested primers^36,46^.

In our study, we selected 14 microsatellite loci amplified in three multiplex reactions, which are useful for further analysis of the breeding biology and population genetics of the European Robin. The criteria of loci selection concentrated on tri- and tetra-nucleotide microsatellites, as they were reported to be less biased with amplification errors, especially stuttering, although they were less polymorphic and frequent than di-nucleotides. However, such an approach as ours results in far fewer problems in reading, and also later coding of alleles^43,46^.

The results of the analysis of nearly 70 unrelated birds from Poland and Portugal showed a high level of polymorphisms of the identified microsatellites, indicated by the values of allelic diversity and heterozygosity. Such a set of markers should allow adequate investigation of genetic diversity and population structure in the European Robin, complementing and advancing on the work of previous studies^22,23^. Indeed, we showed that the identified microsatellites amplified well in the two populations that were separated by a large distance across Europe (c. 2730 km). Comparison of birds from these two populations showed that resident birds from Portugal have slightly lower genetic diversity (in terms of heterozygosity, as well as allelic diversity) than the migratory population from Poland.

Higher genetic diversity of migratory populations compared to resident ones has also been confirmed in North American Golden‑crowned Kinglets (*Regulus satrapa*)^47^.

We showed that genetic differentiation was low between the two populations, yet it was statistically significant. This supports previous studies that showed significant differentiation even within a limited geographical area, such as approximately 100 km^2^ in one example^23^. However, several other studies have suggested that migratory birds have weakly marked genetic differentiation, even over a large area^48–50^. Due to limited geographical sampling so far, the issue of genetic differentiation among populations of European Robins should be investigated further. In particular, for such a widespread and generally abundant species, it would be informative to investigate the relationships and genetic diversity across the very large western Palearctic range, where some populations are highly migratory and others are sedentary or highly insular (e.g. island populations)^1^.

In the analysis of the genotypes of the seven full families of European Robins from Białowieża, we found strong evidence of extra-pair paternity in one nest. Although the sample size was low in this initial study (seven families, 48 nestlings), the result suggests a potentially low frequency of EPP in the study population (15% of sampled families, 4% of nestlings). However, this observation should be verified in a more extensive study, preferably by sampling a large number of representative families and populations across the species’ range.

The rate of EPP that we found for European Robins was within the range of other European forest songbirds, such as the Wood Warbler *Phylloscopus sibilatrix* and Willow Warblers *P. trochilus*, which vary between zero and c. 50% of nests^51–54^. These rates are also similar to many other bird species^55^. Determining rates of EPP can be important for understanding strategies of breeding biology, settlement patterns and mate selection^56–58^.

In summary, our results are the first to provide the essential basis for similar ecological and genetic studies of European Robins, which is an iconic and culturally significant species in parts of Europe^4^, but has nevertheless been poorly studied across much of its range until the present time. We encourage further studies of the genetic diversity of European Robins across its broad geographical range, which may be valuable in understanding any differences between migratory and sedentary populations. Analysis of genetic diversity may also assist in the understanding of any differences between populations inhabiting the varying climatic spectrum in the European Robin’s range (Mediterranean to Boreal) and the wide variety of modified habitats compared to the primeval forest of Białowieża. Comparisons in the genetic variability between other species across this range of enviornments and behaviours would also be valuable.

## Supplementary Information

Supplementary Table S1.
